# De-Implementation of Low-Value Care for Women 70 Years of Age or Older with Low-Risk Breast Cancer During the COVID-19 Pandemic

**DOI:** 10.1245/s10434-023-14156-1

**Published:** 2023-08-25

**Authors:** Ton Wang, Christina Weed, Joshua Tseng, Alice Chung, Marissa K. Boyle, Farin Amersi, Jaswinder Jutla, Amin Mirhadi, Armando E. Giuliano

**Affiliations:** 1 Department of Surgery, Cedars-Sinai Medical Center, Los Angeles, CA USA; 2 Department of Radiation Oncology, Cedars-Sinai Medical Center, Los Angeles, CA USA

## Abstract

**Background:**

Older women with early-stage estrogen receptor-positive (ER+) invasive breast cancer (IBC) are at risk for overtreatment. Guidelines allow for sentinel lymph node biopsy (SLNB) and radiotherapy omission after breast-conserving surgery (BCS) for women 70 years of age or older with T1, clinical node negativity (cN0), and ER+ IBC. The study objective was to evaluate radiotherapy and SLNB de-implementation in older women with low-risk IBC after the resource limitations of the COVID-19 pandemic.

**Methods:**

An institutional database was analyzed to identify women 70 years of age or older who received BCS for IBC from 2012 to 2022. The patients were divided into two cohorts: (1) patients with low-risk IBC (pT1, cN0, and ER+/HER2–) who were eligible for radiotherapy and SLNB omission and (2) patients with high-risk IBC (pT2-T4, cN+, ER–, or HER2+) who were ineligible for therapy omission. Clinicopathologic variables in both cohorts were analyzed.

**Results:**

The study enrolled 881 patients. For the patients with low-risk IBC, the annual rates of radiotherapy were stable from 2012 to 2019. However, radiotherapy utilization decreased significantly from 2020 to 2022 (58% in 2012 vs 36% in 2022; *p* = 0.04). In contrast, radiotherapy usage among the patients with high-risk IBC was stable from 2012 to 2022 (79% in 2012 vs 79% in 2022; *p* = 0.95). Among the patients with low-risk IBC, SLNB rates decreased from 86% in 2012 to 56% in 2022, but this trend predated those in 2020. The factors significantly associated with SLNB and receipt of radiotherapy among the patients with low-risk IBC were younger age, larger tumors, grade 3 disease, and involved nodal status (*p* < 0.01).

**Conclusion:**

This study demonstrated appropriate and sustained de-escalation of radiotherapy in older women with low-risk IBC after the COVID-19 pandemic.

Estimates suggest that more than 30% of U.S. health care expenditure is spent on low-value care, which accounts for more than $100 billion annually.^1^ Overtreatment and low-value care are broadly defined as services that provide minimal benefit to patients but have the potential to result in unnecessary harms and costs. Given the substantial benefits associated with eliminating overtreatment, there is significant interest in identifying the barriers and facilitators to the de-implementation of low-value care.

Early-stage breast cancer is an ideal target for studying the de-implementation of low-value care given its rich history of successive clinical trials demonstrating excellent long-term results after the de-escalation of surgical and adjuvant breast cancer treatment.^2^ Most efforts to de-escalate breast cancer treatment have focused on women 70 years of age or older, who account for approximately one third of all patients with newly diagnosed disease.^3^ The diagnosis for more than 80% of these patients is hormone receptor-positive (HR+), human epidermal growth factor receptor 2 (HER-2)/neu-negative (HER2–) breast cancers with favorable tumor biologies, resulting in estimated 10-year breast-cancer specific survival rates higher than 98%.^3,4^ At the same time, however, older patients are more likely to have significant medical comorbidities and are at the highest risk of harm related to overtreatment.

Based on data from two randomized clinical trials (Cancer and Leukemia Group B 9343 [CALGB 9343] and Post-Operative Radiotherapy in Minimum-Risk Elderly II [PRIME II]) that failed to show a survival benefit associated with adjuvant radiation for older women with low-risk invasive breast cancer (IBC), the National Comprehensive Cancer Network (NCCN) since 2004 has allowed for the omission of adjuvant radiotherapy after breast-conserving surgery (BCS) for women 70 years of age or older with estrogen receptor-positive (ER+), clinically node-negative (cN0) T1 tumors who receive adjuvant endocrine therapy.^5–9^ Similarly, the Society of Surgical Oncology (SSO) and the Choosing Wisely initiative since 2016 have recommended against routine sentinel lymph node biopsy (SLNB) for women 70 years of age or older with cN0 early-stage HR+ IBC.^10^ This recommendation is based on retrospective data and interpretation of CALGB 9343, in which more than 60% of women did not receive axillary staging, with no impact on survival outcomes.^6,11^ Despite these long-standing recommendations, national data demonstrate that more than 80% of women meeting the criteria for SLNB omission and 65% of women meeting the criteria for radiotherapy omission continue to receive these therapies, with minimal change in utilization patterns over time.^12,13^

At the start of the COVID-19 pandemic in early 2020, experts speculated that the resource-limited environment in the acute phase of the pandemic had the potential to eliminate low-value health care.^14^ There were national efforts from multiple organizations to highlight the harms associated with overtreatment and to prioritize the re-distribution of health care utilization from low-value to high-value targets.^15,16^ However, very little data exist to show whether any sustained changes in the de-implementation of low-value care breast cancer care occurred due to these efforts.

The objectives of this study were to determine whether the rates of SLNB and adjuvant radiotherapy for women 70 years of age or older with low-risk breast cancer have been reduced since 2020 and to identify the factors associated with SLNB and radiotherapy utilization for patients eligible for omission of these therapies.

## Methods

### Study Overview

A prospective institutional database registry of breast cancer patients who received multidisciplinary care was reviewed. The study enrolled all women 70 years of age or older with IBC who received BCS between January 2012 and September 2022. The study excluded patients who initially received BCS but subsequently underwent a mastectomy for tumor-involved margins before initiation of adjuvant therapies and patients who received neoadjuvant chemotherapy or neoadjuvant endocrine therapy.

The patients were divided into two cohorts based on their eligibility for omission of SLNB and adjuvant radiotherapy per NCCN and Choosing Wisely guidelines. The low-risk cohort comprised patients with cN0, pathologic T1, ER+, and HER2– IBC who were eligible for omission of axillary staging and radiotherapy. Although Choosing Wisely does not specify T stage or HER2– status as part of their recommendation for SLNB omission, the criteria provided by NCCN for radiotherapy omission were applied to identify a single low-risk cohort that would be eligible for omission of both therapies. The high-risk cohort comprised patients with clinically node-positive (cN+), pathologic T2-T4, triple-negative, or HER2+ IBC who were not eligible for de-escalation of axillary staging and radiotherapy.

Demographic and clinical information including patient age, race, tumor size, clinical and pathologic T and N stages, tumor grade, and histology, as well as ER, progesterone receptor (PR), and HER2 receptor status were evaluated. Treatment information including type of axillary operation (SLNB vs axillary lymph node dissection [ALND]) and receipt of adjuvant chemotherapy, radiotherapy, or endocrine therapy were determined. Due to the contemporary timeline of this study, only initiation of endocrine therapy was evaluated.

The primary study outcome was utilization of axillary staging and adjuvant radiotherapy among women70 years of age or older who received BCS for IBC from 2012 to 2022. The secondary outcomes were patient and tumor factors associated with the utilization of SLNB and radiotherapy for women with low-risk breast cancer who were eligible for omission of these adjuvant therapies. All research was performed after institutional review board approval.

### Statistical Methodology

The demographic and clinical characteristics of the patients with low-risk tumors who were eligible for SLNB and radiotherapy omission were compared with those of the patients with high-risk tumors who were not eligible for omission of these therapies using a two sample *t* test, Fisher’s exact test, or a chi-square test as appropriate. Similar analyses were performed to compare the characteristics of the patients eligible for SLNB and radiotherapy omission who received SLNB, radiotherapy, or both compared with those who did not. Multivariate logistic regression with control for patient and tumor factors was performed to independently evaluate the impact that year of operation had on receipt of axillary staging and adjuvant radiotherapy in both the low- and high-risk patient cohorts. Multivariate logistic regression also was used to examine factors independently associated with receipt of SLNB or adjuvant radiotherapy by those eligible for omission of these therapies.

All statistical analyses were performed using STATA 16.1 (StataCorp LLC, College Station, TX), and two-sided *p* values lower than 0.05 were considered statistically significant.

## Results

### Overall Characteristics

During the study period, 881 women 70 years of age or older received BCS. The low-risk cohort consisted of 520 patients who were eligible for omission of axillary staging and adjuvant radiotherapy. The high-risk cohort had 361 patients who were not eligible for de-escalation of axillary staging and adjuvant radiotherapy.

The characteristics of the patients who were eligible for omission of these therapies compared with those who were not are shown in Table 1. The high-risk cohort differed significantly in terms of patient age, race, tumor grade, and histology, and had more elderly patients, black patients, and patients with grade 3 and lobular IBC who were not eligible for omission of axillary staging and radiotherapy (*p* ≤ 0.03). Overall, 64.7% of the patients eligible for omission received SLNB compared with 74.8% of the patients not eligible for omission (*p* <0.01).

**Table 1:** Characteristics of women ≥70 years old who received BCS for invasive breast cancer in the low-risk cohort (cN0, T1, ER+, HER2– invasive breast cancer) who were eligible for omission of axillary staging and adjuvant radiotherapy versus women in the high-risk cohort (pT2-T4, cN+, ER–, or HER2+) who were not eligible for omission of these therapies

	Eligible for omission (*n* = 520) *n* (%)^a^	Not eligible for omission (*n* = 361) *n* (%)^a^	*p* Value
Mean age (years)	76.3 ± 5.2	78.0 ± 6.6	**< 0.01**
Age group (years)			**< 0.01**
70–75	267 (51.4)	170 (47.1)	
76–80	143 (27.5)	84 (23.3)	
81–85	81 (15.6)	49 (13.6)	
≥86	29 (5.6)	58 (16.1)	
Race			**< 0.01**
White	435 (83.7)	274 (75.9)	
Black	42 (8.1)	60 (16.6)	
Asian	31 (6.0)	18 (5.0)	
Other	12 (2.3)	9 (2.5)	
Clinical T stage			–
cTis	25 (4.8)	7 (1.9)	
cT1	443 (85.2)	177 (49.0)	
cT2	39 (7.5)	154 (42.7)	
cT3	0 (0.0)	6 (1.7)	
cT4	0 (0.0)	5 (1.4)	
cTx	13 (2.5)	12 (3.3)	
Clinical N stage			–
cN0	520 (100.0)	325 (90.0)	
cN1	0 (0.0)	26 (7.2)	
cN2	0 (0.0)	2 (0.6)	
cNx	0 (0.0)	8 (2.2)	
Pathologic T stage			–
pT1	520 (100.0)	99 (27.4)	
pT2	0 (0.0)	242 (67.0)	
pT3	0 (0.0)	16 (4.4)	
pT4	0 (0.0)	4 (1.1)	
Pathologic N stage			–
pN0	299 (57.5)	175 (48.5)	
pN1	36 (6.9)	69 (19.1)	
pN2	1 (0.2)	20 (5.5)	
pN3	0 (0.0)	6 (1.7)	
pNX	184 (35.4)	91 (25.2)	
Grade			**< 0.01**
1	138 (26.5)	31 (8.6)	
2	300 (57.7)	179 (49.6)	
3	69 (13.3)	148 (41.0)	
Unknown	13 (2.5)	3 (0.8)	
Mean tumor size (mm)	11.1 ± 5.0	26.3 ± 14.3	–
Histology			**0.03**
Ductal	388 (74.6)	264 (73.1)	
Lobular	57 (11.0)	55 (15.2)	
Mixed ductal and other	63 (12.1)	28 (7.8)	
Other	12 (2.3)	14 (3.9)	
ER status			–
Positive	520 (100.0)	266 (73.7)	
Negative	0 (0.0)	95 (26.3)	
PR status			–
Positive	469 (90.2)	245 (67.9)	
Negative	51 (9.8)	116 (32.1)	
HER2 status			–
Positive	0 (0.0)	66 (18.3)	
Negative	520 (0.0)	292 (80.9)	
Equivocal	0 (0.0)	3 (0.8)	
Year of operation			0.46
2012	28 (5.4)	26 (7.2)	
2013	26 (5.0)	25 (6.9)	
2014	43 (8.3)	33 (9.1)	
2015	28 (5.4)	27 (7.5)	
2016	56 (10.8)	39 (10.8)	
2017	57 (11.0)	27 (7.5)	
2018	53 (10.2)	40 (11.1)	
2019	66 (12.7)	47 (13.0)	
2020	62 (11.9)	30 (8.3)	
2021	66 (12.7)	44 (12.2)	
2022	35 (6.7)	23 (6.4)	
Axillary staging			**< 0.01**
SLNB	331 (63.7)	226 (62.6)	
ALND	5 (1.0)	44 (12.2)	
None	184 (35.4)	91 (25.2)	
Nodal status			**< 0.01**
Tumor-involved	37 (11.0)	95 (35.2)	
Tumor-free	299 (89.0)	175 (64.8)	
Chemotherapy			**< 0.01**
Yes	20 (3.9)	91 (25.2)	
No	500 (96.2)	270 (74.8)	
Radiotherapy			**< 0.01**
Yes	265 (51.0)	241 (66.8)	
No	255 (49.0)	120 (33.2)	
Unknown			
Endocrine therapy^b^			0.35
Yes	430 (82.7)	209 (78.6)	
No	89 (17.1)	56 (21.1)	
Unknown	1 (0.2)	1 (0.4)	

The bold numbers are used to highlight variables that are statistically significant (*p* < 0.05)

*BCS* breast-conserving surgery; *ER* estrogen receptor; *HER2* human epidermal growth factor receptor 2; *SLNB* sentinel lymph node biopsy; *ALND* axillary lymph node biopsy

^a^Percentages reported by column

^b^Endocrine therapy if ER+

Among the patients who received axillary staging, 11% of the patients eligible for omission had tumor-involved lymph nodes, compared with 35.2% of the patients who were not eligible for omission of axillary staging (*p* < 0.01). Of the patients eligible for omission, 51% received adjuvant radiotherapy compared with 66.8% of the patients who were not eligible for omission (*p* < 0.01). Significantly more patients in the high-risk cohort received treatment with chemotherapy than patients in the low-risk cohort (25.2% vs 3.9%; *p* < 0.01). The ER+ patients in the two cohorts did not differ in receipt of endocrine therapy.

### Trends in Radiotherapy Utilization

**Fig. 1:**
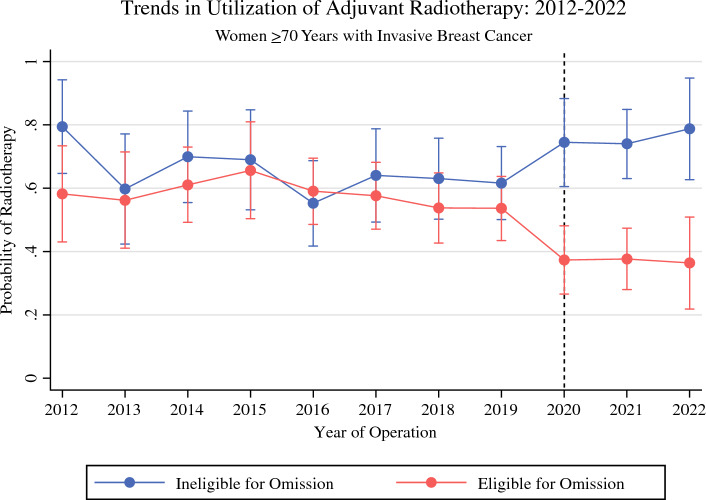
Predicted probability of adjuvant radiotherapy from 2012 to 2022 with multivariate adjustment for women ≥70 years old who received breast-conserving surgery (BCS) for invasive breast cancer

The rates of adjuvant radiotherapy among the women 70 years of age or older who received BCS for IBC from 2012 to 2022 are shown in Fig. 1. The adjusted probability of radiotherapy receipt for the patients in the low-risk cohort who were eligible for radiotherapy omission was stable from 2012 to 2019, ranging from 54 to 66%. However, the probability of radiotherapy utilization decreased significantly from 2020 to 2022 (range, 36–38%). Year of operation in 2020–2022 was an independent predictor of decreased odds of radiotherapy receipt (Table 2).

**Table 2:** Adjusted odds ratios (OR) with 95% confidence intervals (CIs) from multivariable logistic regression analysis predicting the odds of receiving SLNB and post-BCS radiotherapy among women ≥70 years old in the low-risk cohort (cN0, T1, ER+, HER2- invasive breast cancer) who were eligible for omission of these therapies

	SLNB OR (95% CI)	Radiotherapy OR (95% CI)
Year of operation		
2012 (reference)	1.00	1.00
2013	0.53 (0.11–2.50)	0.88 (0.21–3.67)
2014	0.80 (0.18–3.56)	1.22 (0.33–4.47)
2015	0.58 (0.11–2.94)	1.69 (0.37–7.64)
2016	0.39 (0.10–1.59)	1.06 (0.31–3.63)
2017	0.35 (0.09–1.43)	0.96 (0.28–3.26)
2018	**0.18 (0.04–0.71)**	0.75 (0.21–2.62)
2019	**0.09 (0.02–0.36)**	0.74 (0.21–2.53)
2020	**0.18 (0.04–0.69)**	**0.26 (0.08–0.90)**
2021	**0.19 (0.05–0.77)**	**0.27 (0.08–0.89)**
2022	**0.16 (0.04–0.68)**	**0.25 (0.06–0.98)**
Age	**0.86 (0.83–0.90)**	**0.80 (0.76–0.85)**
Race		
White (reference)	1.00	1.00
Black	1.14 (0.52–2.48)	1.95 (0.82–4.65)
Asian	0.89 (0.34–2.34)	1.16 (0.39–3.48)
Other	1.17 (0.27–5.10)	1.41 (0.28–7.11)
Tumor size (mm)	**1.09 (1.04–1.15)**	**1.05 (1.00–1.11)**
Grade		
1 (reference)	1.00	1.00
2	1.16 (0.71–1.88)	0.70 (0.40–1.21)
3	1.64 (0.79–3.41)	**2.96 (1.27–6.86)**
Histology		
Ductal (reference)	1.00	1.00
Lobular	1.10 (0.53–2.27)	0.87 (0.41–1.85)
Mixed ductal and other	0.97 (0.47–1.98)	1.08 (0.51–2.28)
Other	0.98 (0.24–4.01)	0.98 (0.20–4.84)
Nodal status		
Tumor-free (reference)	–	1.00
Tumor-involved	–	**4.34 (1.22–15.43)**
Not assessed	–	**0.21 (0.12–0.35)**
Endocrine therapy		
No (reference)	–	1.00
Yes	–	1.56 (0.82–2.99)

The bold numbers are used to highlight variables that are statistically significant (*p* < 0.05)

*SLNB* sentinel lymph node biopsy; *BCS* breast-conserving surgery; *ER* estrogen receptor; *HER2* human epidermal growth factor receptor 2

In contrast, the adjusted probability of radiotherapy receipt for patients in the high-risk cohort who were not eligible for radiotherapy omission did not change significantly from 2012–2019 (range, 55–79%) to 2020–2022 (range, 73–79%). In this cohort, the probability of radiotherapy receipt in 2012 was 79% compared to 79% in 2022, and year of operation was not an independent predictor of odds of radiotherapy receipt (Table 2).

### Trends in SLNB Utilization

**Fig. 2:**
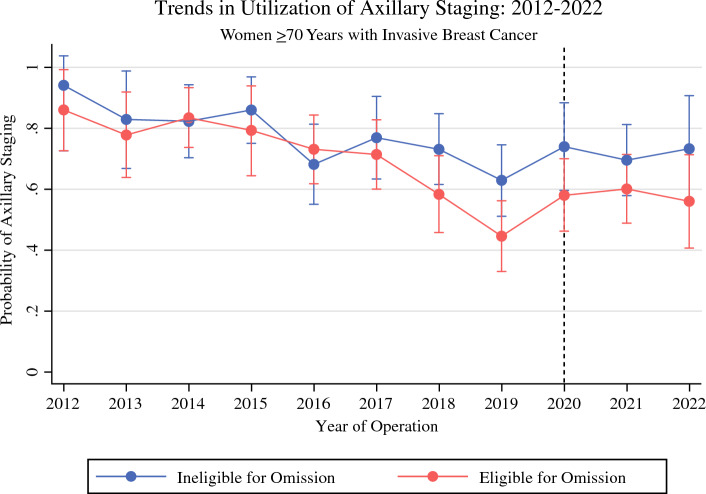
Predicted probability of axillary staging from 2012 to 2022 with multivariate adjustment for women ≥70 years old who received breast-conserving surgery (BCS) for invasive breast cancer

The rates of axillary staging among the women 70 years of age or older who received BCS for IBC from 2012 to 2022 are shown in Fig. 2. During the study period, utilization of axillary staging for the patients in the low-risk cohort showed a significant reduction, with the adjusted probability of axillary staging decreasing from 86% in 2012 to 56% in 2022 (range, 45–86%). However, this trend predated 2020, with year of operation from 2018 to 2022 as an independent predictor of decreased odds of SLNB receipt (Table 2). The high-risk cohort showed a decrease in the adjusted probability of axillary staging, from 94% in 2012 to 73% in 2022 (range, 63–94%). However, the pattern of decrease was less consistent. Compared with 2012, year of operation in 2016, 2018, 2019, and 2021 were independent predictors of decreased odds of axillary staging receipt. For three consecutive years, significant de-escalation was not identified.

### Factors Associated with Radiotherapy and SLNB Utilization Among Patients Eligible for Omission

Table 3 shows univariate analyses comparing clinical characteristics of the patients in the low-risk cohort who received SLNB and adjuvant radiotherapy with those of the patients who did not receive these therapies. Table 2 shows adjusted odds ratios (ORs) from multivariate logistic regression analyses predicting SLNB and adjuvant radiotherapy utilization among the patients in the low-risk cohort who were eligible for omission of these therapies.

**Table 3:** Characteristics of women ≥70 years old in the low-risk cohort (cN0, T1, ER+, HER2– invasive breast cancer) who were eligible for omission of axillary staging and post-BCS radiotherapy by receipt of these therapies

	SLNB (*n* = 336) *n* (%)^a^	No SLNB (*n* = 184) *n* (%)^a^	*p* value^b^	Radiation (*n* = 265) *n* (%)^a^	No radiation (*n* = 255) *n* (%)^a^	*p* value^c^
Year of operation			**< 0.01**			**< 0.01**
2012	25 (89.3)	3 (10.7)		20 (71.4)	8 (28.6)	
2013	19 (73.1)	7 (26.9)		14 (53.9)	12 (46.2)	
2014	33 (76.7)	10 (23.3)		24 (55.8)	19 (44.2)	
2015	23 (82.1)	5 (17.9)		21 (75.0)	7 (25.0)	
2016	43 (76.8)	13 (23.2)		38 (67.9)	18 (32.1)	
2017	42 (73.7)	15 (26.3)		35 (61.4)	22 (38.6)	
2018	29 (54.7)	24 (45.3)		27 (50.9)	26 (49.1)	
2019	28 (42.4)	38 (57.6)		30 (45.5)	36 (54.6)	
2020	34 (54.8)	28 (45.2)		20 (32.3)	42 (67.7)	
2021	42 (63.6)	24 (36.4)		26 (39.4)	40 (60.6)	
2022	18 (51.4)	17 (48.6)		10 (28.6)	25 (71.4)	
Mean age (years)	75.1 ± 4.5	78.6 ± 5.6	**< 0.01**	74.2 ± 3.8	78.6 ± 5.4	**< 0.01**
Age group (years)			**< 0.01**			**< 0.01**
70–75	205 (76.8)	62 (23.2)		180 (67.4)	87 (32.6)	
76–80	90 (62.9)	53 (37.1)		66 (46.2)	77 (53.9)	
81–85	32 (39.5)	49 (60.5)		16 (19.8)	65 (80.3)	
≥86	9 (31.0)	20 (69.0)		3 (10.3)	26 (89.7)	
Race			0.91			0.21
White	280 (64.4)	155 (35.6)		213 (49.0)	222 (51.0)	
Black	29 (69.1)	13 (31.0)		26 (61.9)	16 (38.1)	
Asian	19 (61.3)	12 (38.7)		18 (58.1)	13 (41.9)	
Other	8 (66.7)	4 (33.3)		8 (66.7)	4 (33.3)	
Mean tumor size (mm)	11.9 ± 4.8	9.7 ± 4.9	**< 0.01**	12.0 ± 4.9	10.2 ± 4.9	**< 0.01**
Grade			**< 0.01**			**< 0.01**
1	79 (57.3)	59 (42.8)		61 (44.2)	77 (55.8)	
2	202 (67.3)	98 (32.7)		150 (50.0)	150 (50.0)	
3	51 (73.9)	18 (26.1)		49 (71.0)	20 (29.0)	
Unknown	4 (30.8)	9 (69.2)		5 (38.5)	8 (61.5)	
Histology			0.14			0.28
Ductal	244 (62.9)	144 (37.1)		194 (50.0)	194 (50.0)	
Lobular	38 (66.7)	19 (33.3)		29 (50.9)	28 (49.1)	
Mixed ductal and other	48 (76.2)	15 (23.8)		38 (60.3)	25 (39.7)	
Other	6 (50.0)	6 (50.0)		4 (33.3)	8 (66.7)	
Axillary staging			–			**< 0.01**
Yes	–	–		229 (68.2)	107 (31.9)	
No	–	–		36 (19.6)	148 (80.4)	
Nodal status^d^			–			**< 0.01**
Tumor-involved	37 (11.0)	–		34 (91.9)	3 (8.1)	
Tumor-free	299 (89.0)	–		196 (65.2)	104 (34.8)	
Chemotherapy			0.97			**< 0.01**
Yes	13 (65.0)	7 (35.0)		18 (90.0)	2 (10.0)	
No	323 (64.6)	177 (35.4)		247 (49.4)	253 (50.6)	
Radiotherapy			**< 0.01**			–
Yes	229 (86.4)	36 (13.6)		–	–	
No	107 (42.0)	148 (58.0)		–	–	
Endocrine therapy			**< 0.01**			**< 0.01**
Yes	296 (68.8)	134 (31.2)		237 (55.1)	193 (44.9)	
No	39 (43.8)	50 (56.2)		27 (30.3)	62 (69.7)	
Unknown	1 (100.0)	0 (0.0)				

The bold numbers are used to highlight variables that are statistically significant (*p* < 0.05)

*ER* estrogen receptor; *HER2* human epidermal growth factor receptor 2; *BCS* breast-conserving surgery; *SLNB* sentinel lymph node biopsy

^a^Percentages reported by row

^b^*p* value comparing characteristics of patients who received axillary staging versus patients who omitted axillary staging

^c^*p* value comparing characteristics of patients who received radiotherapy versus patients who omitted radiotherapy

^d^If axillary staging was performed

In both the uni- and multivariate analyses, older age was associated with significantly decreased odds for receipt of SLNB (OR, 0.86; 95% confidence interval [CI], 0.83–0.90) or radiotherapy (OR, 0.80; 95% CI, 0.76–0.85), whereas increased tumor size was associated with significantly increased odds for receipt of SLNB (OR, 1.09; 95% CI, 1.04–1.15) or radiotherapy (OR, 1.05; 95% CI, 1.00–1.11). Specifically, the patients in age categories of 70 to 75 years and 76 to 80 years were more likely to receive SLNB, whereas the patients 81 years old or older were more likely to omit SLNB (*p* < 0.01). The patients 70 to 75 years old were more likely to receive radiotherapy, whereas the patients 76 years or older were more likely to omit adjuvant radiotherapy (*p* < 0.01). The univariate analyses showed that the patients with grade 3 disease were more likely to receive both axillary staging and radiotherapy (*p* < 0.01), although the multivariate analyses showed that grade 3 disease was associated only with significantly higher odds of radiotherapy receipt (OR, 2.96; 95% CI, 1.27–6.86) and not SLNB receipt (OR, 1.64; 95% CI, 0.79–3.41). There was no difference in utilization of axillary staging (*p* = 0.14) or radiotherapy (*p* = 0.28) based on tumor histology.

The univariate analyses showed that significantly more patients who received SLNB were treated with radiotherapy and endocrine therapy (*p* < 0.01), but chemotherapy receipt did not differ between the patients who received axillary staging and those who did not (*p* = 0.97). The multivariate analyses showed that the patients with tumor-involved nodes were at significantly higher odds of radiotherapy receipt (OR, 4.34; 95% CI, 1.22–15.43), whereas the patients who did not receive axillary staging had significantly lower odds of radiotherapy receipt (OR, 0.21; 95% CI, 0.12–0.35). The univariate analyses showed that significantly more patients who underwent radiotherapy were treated with chemotherapy and endocrine therapy (*p* < 0.01). However, the adjusted multivariate analyses showed that receipt of endocrine therapy was not associated with increased odds of radiotherapy utilization (OR, 1.56; 95% CI, 0.82–2.99).

## Discussion

This study demonstrated significant de-implementation of adjuvant radiotherapy among women 70 years of age or older with ER+, HER2–, cN0, T1 IBC after the start of the COVID-19 pandemic in 2020. Among these patients with low-risk breast cancer, adjuvant radiotherapy is associated with a small reduction in locoregional recurrence, but no survival benefit.^6,8^ Among patients eligible for omission of post-BCS radiotherapy, the probability of radiotherapy receipt decreased by approximately one third from 2020–2022 to 2012–2019.

Although the retrospective nature of this study did not allow for the direct determination of a causal relationship between the COVID-19 pandemic and radiotherapy utilization, the abrupt de-implementation of adjuvant radiotherapy among eligible patients in 2020 supports an effect from the COVID-19 pandemic rather than more generalized efforts at de-escalation of breast cancer treatment. Specifically, resource limitations during the acute phase of the pandemic increased awareness about the benefits of avoiding low-value care. This was supported by the April 2020 COVID-19 Pandemic Breast Cancer Consortium expert consensus, which explicitly recommended omission or deferral of radiotherapy for patients 65 to 70 years of age or older with early-stage ER+ IBC.^16^ Before 2020, the rates of radiotherapy in this study remained stable despite several opportunities for de-implementation during the last decade, including release of 10-year data from CALGB 9343 in 2013, inclusion of low-value breast cancer care as a focus of the Choosing Wisely campaign in 2016, and release of the results from PRIME II in 2017.

In contrast to the de-escalation of adjuvant radiotherapy among the patients with low-risk breast cancer, there was no change in rates of radiotherapy delivery to patients who were not candidates for omission during the study period. Although the COVID-19 pandemic in 2020 complicated the delivery of radiotherapy, the study institution was able to provide continued high rates of radiotherapy to patients with high-risk breast cancer who required adjuvant treatment. These practice patterns, with targeted de-implementation of radiotherapy for patients with low-risk breast cancer and simultaneous high rates of radiotherapy for patients with high-risk breast cancer, persisted for at least 2 years after the acute phase of the pandemic without rebound to pre-pandemic patterns. This suggests sustained changes in clinical practice patterns and provider comfort with not recommending radiotherapy for patients at low risk for locoregional recurrence.

Although the rates of SLNB among the patients in the low-risk cohort decreased significantly from 2012 to 2022, this trend predated 2020 and did not appear to have been affected by the COVID-19 pandemic. Additionally, the rates of SLNB among the patients in the high-risk cohort also decreased during the study period, although to a lesser extent. This finding likely was related to several factors. First, this study restricted eligibility for SLNB omission to patients with T1 tumors in order to define a low-risk cohort eligible for both radiotherapy and SLNB omission. However, SSO and Choosing Wisely both recommend SLNB omission for patients with “early-stage cancer,” which can be interpreted by providers to include larger tumors.

Second, the decision to perform SLNB is based on clinical stage. In circumstances in which SLNB is initially omitted, providers may not elect to return to the operating room for axillary staging if a tumor was upstaged on final pathology.

Finally, in 2016, the study institution piloted a clinical trial evaluating the safety and efficacy of SLNB omission for patients 65 years of age or older with ER+, HER2–, T1-T2 IBC. The patients with T2 tumors in this trial for whom SLNB was omitted would have been classified as part of the high-risk cohort who were ineligible for SLNB omission in the current study.

The finding that radiotherapy and SLNB utilization were affected differently by the COVID-19 pandemic likely reflects discrepancies in provider attitudes on the potential harms of these two services. Although radiotherapy requires significant utilization of health care resources and risks exposure of both patients and providers to COVID-19 infection, performing SLNB at the time of a planned breast cancer operation may appear relatively benign to surgeons. This distinction is also reflected in the April 2020 recommendations by the COVID-19 Pandemic Breast Cancer Consortium. Whereas there is specific guidance to omit or defer radiotherapy for patients 65 to 70 years of age or older with early-stage ER+ IBC, there is no recommendation to avoid axillary staging for this patient population.^16^ Prior qualitative research evaluating provider barriers to de-implementation of low-value SLNB demonstrated that surgeons view SLNB as an easy and low-risk procedure.^17^ However, despite this bias, SLNB is not benign. Rates of lymphedema after SLNB are approximately 5%, and studies have shown that patients who receive axillary staging are significantly more likely to receive adjuvant radiotherapy despite their potential as appropriate candidates for omission of both low-value therapies.^18,19^ This was supported by the current study, which found that the patients in the low-risk cohort who received SLNB, even if found to have tumor-free lymph nodes, were significantly more likely to receive adjuvant radiotherapy. The cascade effect, in which one low-value procedure leads to increased utilization of other low-value therapies, is a described phenomenon that exists across medical subspecialities and has been shown to result in significant harm to patients, health care costs, and emotional distress.^20,21^

Predictably, the factors associated with increased odds of SLNB and radiotherapy utilization among the patients in the low-risk cohort included younger age, larger tumors, high-grade tumors, and tumor-involved lymph nodes. The finding that grade 3 disease was significantly associated with receipt of radiotherapy among patients otherwise candidates for omission is interesting because it reflects a general concern among radiation oncologists that patients with grade 3 tumors were underrepresented in CALGB 9343 and PRIME II.^22^ In the findings of CALGB 9343, tumor grade was not reported, and only 3% of the patients in PRIME II had grade 3 tumors. This discrepancy likely was due in part to the overall low-risk biology of small ER+ breast cancers in women 70 years of age or older. Only 13% of the patients in this study who were eligible for radiotherapy omission had grade 3 disease compared with 41% of the patients who were not eligible for omission. However, high-grade disease is known to be one of the strongest independent risk factors for locoregional recurrence, and among these patients, radiotherapy administration is associated with substantial reductions in recurrence risk.^23^ Thus, radiotherapy for patients with grade 3 tumors may be beneficial even for older patients with early-stage, ER+/HER2– disease.

The finding that nodal involvement is significantly associated with increased rates of radiotherapy is frequently cited as a reason not to de-escalate SLNB, because the nodal status may have an impact on what adjuvant therapies a patient receives.^24^ However, in the modern era of molecular testing, the importance of pathologic staging has diminished. The RxPonder trial demonstrated that the benefit of chemotherapy for post-menopausal women with ER+ IBC depends on tumor biology, and thus the Oncotype Dx score rather than the identification of nodal metastases now largely dictates whether a patient receives chemotherapy.^25^ The potential benefit of adjuvant radiotherapy for women 70 years of age or older with ER+/HER2– IBC who have tumor-involved lymph nodes is of greater debate. However, it is worth noting that although two thirds of the patients in CALGB 9343 did not receive nodal staging, the 10-year locoregional recurrence rate was lower than 10%, and the rate of axillary recurrence was only 3% for the women who omitted radiotherapy and were treated with endocrine therapy alone.^6^ This finding is further supported by data from retrospective studies that have found no difference in survival rates among patients 70 years of age or older with ER+, HER2–, cN0 IBC regardless of radiotherapy receipt if endocrine therapy is administered, even among patients with nodal metastases on SLNB.^26^

Finally, the rates of nodal positivity are low among patients meeting the criteria for omission of axillary staging. In the low-risk cohort, 11% of the patients had sentinel lymph node involvement compared with 35% of the patients in the cohort that was not eligible for omission of axillary staging. This is supported by other studies that have found the rate of nodal involvement among women 70 years of age or older with cN0, T1, ER+/HER2– IBC to be lower than 10%.^27^

The target rate for SLNB and radiotherapy de-implementation among patients eligible for omission of these therapies is unknown. However, the dramatic decrease in rates of completion ALND among patients with one or two tumors involving lymph nodes who received BCS and radiotherapy after dissemination of the ACOSOG Z0011 trial can serve as a model for successful de-implementation.^28^ National data suggest that among patients meeting eligibility criteria for Z0011, the rates of completion ALND decreased from 63% in 2004 to 14% in 2016, a relative reduction of 78%.^13^ Thus, despite the significant de-implementation of radiotherapy and SLNB among women 70 years of age or older with low-risk ER+ IBC observed in the current study, opportunity likely exists for continued de-escalation.

Prior work has shown that several factors are associated with utilization of low-value SLNB and radiotherapy for breast cancer patients. First, patient age is strongly correlated with SLNB and radiotherapy receipt among patients who are candidates for omission.^12,29^ This is supported by findings in the current study showing that few patients older than 80 years with low-risk ER+ IBC received SLNB or radiotherapy.

Second, both patients and providers refer to the importance of considering physiologic age in addition to biologic age when age-based guidelines are evaluated. Qualitative work has shown that patients who feel healthier than average are more likely to desire aggressive care despite recommendations for omission.^30^ Similarly, surgeons frequently describe making treatment decisions based on a patient’s functional status rather than a patient’s biologic age.^17^ However, despite the bias that healthy patients with low-risk ER+ IBC might benefit more from SLNB and radiotherapy, it is important to note that the patients included in CALGB 9343 had a higher overall survival than age-matched women in the general population, suggesting that the results of this trial apply equally to women who are healthier than average and without significant comorbid conditions.^6^

Finally, patient desire to pursue more versus less medical care may be an inherent personal trait. This concept has been shown through the medical maximizer-minimizer scale, in which “medical maximizers“ tend to elect for health care interventions in situations in which it may not be necessary, whereas “medical minimizers” tend to avoid health care inventions unless they are absolutely necessary.^31^ The correlation between medical maximizer-minimizer preferences and receipt of SLNB and radiotherapy among women with low-risk breast cancer has previously been shown.^32,33^ The tendency for medical maximizers to want “everything done” may explain, at least in part, why patients with low-risk IBC who received SLNB were more likely to receive radiotherapy even in the absence of nodal positivity.

This study was limited by its single-institution nature, so these findings may not be representative of national practice patterns. However, post-pandemic data from national databases such as the National Cancer Database (NCDB) and the Surveillance, Epidemiology, and End Results (SEER) program will not be available for analysis for several years. Additionally, due to the contemporary nature of this study, there are no long-term data on whether patients for whom radiotherapy was omitted completed their recommended course of adjuvant endocrine therapy. However, the decision to omit radiotherapy is based on clinical judgment as to whether a patient is likely to tolerate adjuvant endocrine therapy, so evaluating initiation of endocrine therapy is likely an appropriate surrogate.

Patient age and tumor characteristics alone do not capture the nuances involved in the decision for a patient to omit or receive axillary staging and radiotherapy. The decision relies on shared decision-making regarding the potential risks versus the benefits of these therapies, and not every patient who has been identified in the low-risk cohort may be truly appropriate for omission of radiotherapy and SLNB.

Finally, factors other than the COVID-19 pandemic may have affected the trends observed in this study. However, the sharp change in radiotherapy practice patterns in 2020 with little to no change in the years before suggests a strong correlation with the COVID-19 pandemic.

In conclusion, this study demonstrated appropriate de-escalation of adjuvant radiotherapy for women 70 years of age or older with cN0, T1, ER+, HER2– IBC during the COVID-19 pandemic without a change in rates of radiotherapy receipt among patients with high-risk breast cancer. This reflects intentional, evidence-based change in provider practice patterns to avoid low-value care, which has been sustained over time. Although there has been de-implementation of SLNB for patients eligible for omission of axillary staging since 2012, this trend appears to predate 2020 and did not change with the COVID-19 pandemic. This demonstrates that reduction in low-value care offers an opportunity for dissemination of strategies to reduce overtreatment at other institutions and at a national level.
